# Nanophotonics shines light on hyperbolic metamaterials

**DOI:** 10.1038/s41377-021-00688-2

**Published:** 2022-01-10

**Authors:** Andreas Aigner, Judith M. Dawes, Stefan A. Maier, Haoran Ren

**Affiliations:** 1 Chair in Hybrid Nanosystems, Nanoinstitute Munich, Faculty of Physics, Ludwig-Maximilians-University Munich, Munich, 80539 Germany; 2 MQ Photonics Research Centre, Department of Physics and Astronomy, Macquarie University, Macquarie Park, NSW 2109 Australia; 3 Department of Physics, Imperial College London, London, SW7 2AZ UK

**Keywords:** Metamaterials, Micro-optics

## Abstract

Hyperbolic metamaterials with a unique hyperbolic dispersion relation allow propagating waves with infinitely large wavevectors and a high density of states. Researchers from Korea and Singapore provide a comprehensive review of hyperbolic metamaterials, including artificially structured hyperbolic media and natural hyperbolic materials. They explain key nanophotonic concepts and describe a range of applications for these versatile materials.

Metamaterials, consisting of subwavelength meta-atoms (artificially designed unit cells of metamaterials), exhibit anomalous optical properties that cannot be found in naturally occurring materials. In the metamaterials family, hyperbolic metamaterials (HMMs) have attracted strong interest due to their unique and extreme anisotropy that renders the metamaterials to behave like a metal in one direction and a dielectric in the other. Writing in *eLight*, Dasol Lee, Sunae So, Guangwei Hu, Cheng-Wei Qiu, Junsuk Rho, and colleagues now provide a comprehensive review on HMMs from fundamentals to applications, covering both artificial metamaterials and recently discovered natural 2D materials that support hyperbolic dispersion^1^.

HMMs are named after the topology of their isofrequency contour. In vacuum, the linear dispersion and isotropic behavior of propagating waves implies a spherical isofrequency contour given by the equation

1 $$k_x^2 + k_y^2 + k_z^2 = w^2/c^2$$

where the wavevector of a propagating wave is given by $\vec k = [k_x,k_y,k_z]$, *w* is the frequency of radiation and *c* is the velocity of light in free space. The dispersion relation of light in a medium with an effective permittivity tensor $\vec \varepsilon = [\varepsilon _{xx},\varepsilon _{yy},\varepsilon _{zz}]$ leads to

2 $$\frac{{k_x^2 + k_y^2}}{{\varepsilon _{zz}}} + \frac{{k_z^2}}{{\varepsilon _{xx}}} = w^2/c^2$$

where the in-plane isotropic components are *ε*_*xx*_ = *ε*_*yy*_ and out-of-plane component is *ε*_*zz*_. In common materials, the isofrequency contour is represented by a spherical surface (Fig. 1a) for the isotropic case, or an ellipsoid (Fig. 1b) for the anisotropic case, respectively. However, under the condition where the signs of two components of the effective permittivity are opposite $\left( {\varepsilon _{xx} \cdot \varepsilon _{zz} \,<\, 0} \right)$, the isofrequency contour becomes an unbounded hyperboloid that can support very large magnitude wavevectors^2^. In this case, two classes of HMMs have been identified: Type-I HMMs $\left( {\varepsilon _{xx} = \varepsilon _{yy} \,>\, 0,\varepsilon _{zz} \,<\, 0} \right)$ have a predominantly dielectric nature (Fig. 1c); Type-II HMMs $\left( {\varepsilon _{xx} = \varepsilon _{yy} \,<\, 0,\varepsilon _{zz} \,>\, 0} \right)$ have a metallic nature (Fig. 1d). Type-II HMMs are highly reflective and are more absorptive than Type-I HMMs^3^.

**Fig. 1:**
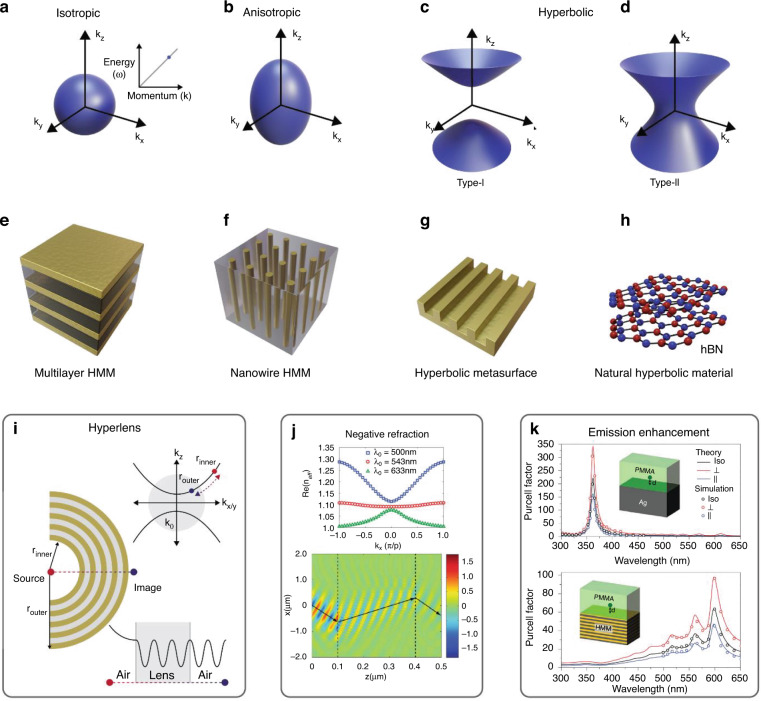
Principles and applications of hyperbolic metamaterials. Isofrequency contour for **a** isotropic, **b** anisotropic media, and **c** Type-I and **d** Type-II hyperbolic materials. Different hyperbolic material designs: **e** multilayer hyperbolic metamaterial (HMM), **f** nanowire HMM, **g** hyperbolic metasurface, and **h** natural hyperbolic material. Different applications of HMMs: **i** a HMM hyperlens, **j** negative refraction in a plasmonic grating HMS, and **k** spontaneous emission enhancement above a silver surface (top) and a HMM (bottom). **i**, **j**, and **k** are adapted from^3,13^, and^16^, respectively

This Review by Lee et al.^1^ highlights different nanophotonic structures that have been developed to support hyperbolic dispersion for HMMs. These structures include multilayer HMM (Fig. 1e), nanowire HMM (Fig. 1f), hyperbolic metasurface (Fig. 1g), and natural hyperbolic materials (Fig. 1h).

Multilayer HMMs (Fig. 1e), consisting of alternating layers of metal and dielectric, have been developed to exhibit extreme anisotropy^4^. If the layer thicknesses are far smaller than the operating wavelength, then effective medium theory can be used to describe the macroscopic optical responses of these composite materials using homogenized effective permittivity tensors. The effective permittivity can be customized by the choice of materials and different thicknesses of metal and dielectric layers. Tuning these two parameters can yield both Type-I and Type-II multilayer HMMs, as well as effective dielectric or metallic properties^5^.

Nanowire HMMs (Fig. 1f) offer another approach to realizing HMMs, where metallic nanowires are embedded in a dielectric host medium^6^. The real part of the metal permittivity is negative at frequencies below the plasma frequency, so the nanowire HMMs can exhibit Type-I HMM behavior without the requirement of an optical resonance. Consequently, they can operate at a relatively low loss and high transmission over a large bandwidth, although the electrochemical deposition method to date has a limited choice of materials^1^.

Metasurfaces have recently transformed device design for nearly arbitrary tailored light control via arrays of ultrathin meta-atoms. Unlike the bulk multilayer HMM that generally features high Ohmic loss and a complicated multistep fabrication process, ultrathin hyperbolic metasurfaces (HMSs) could alleviate these challenges. Metallic HMSs (Fig. 1g) exploit the control of the propagation of surface plasmon polaritons (SPPs) along the metasurface, leading to a hyperbolic dispersion of in-plane SPPs. The signs of the imaginary parts of the effective surface conductivity components are different. Consequently, HMSs can support extremely large wavevectors, as well as a large photonic density of states^7^.

Natural materials can also exhibit hyperbolic dispersion (Fig. 1h). A layered crystal structure can allow 2D conductivity and out-of-plane insulation. Since the fabrication resolution of HMMs is limited, the effective permittivity homogenization model breaks down for small wavelengths and structures close to resonance where losses are increased significantly. Furthermore, multilayer and nanowire HMMs require plasmonic metals, which are always linked with Ohmic losses. Natural HMMs overcome these limitations and can be divided into two classes. First, hyperbolic plasmon polariton materials like black phosphorus^8^ and tungsten ditelluride^9^ in which light and free electrons couple. Hyperbolic phonon polariton (HPhP) materials, like hexagonal boron nitride^10^, represent the second type. They are generally composed of 2D sheets of strongly coupled atoms by covalent bonds with weak van der Waals coupling between the stacked sheets. Then polaritonic coupling between photons and phonons (lattice vibrations) is highly anisotropic and out-of-plane hyperbolicity can occur. In contrast to plasmonic systems, HPhP materials are immune to Ohmic losses due to the absence of electron-electron scattering^10^. The operating wavelength range occurs in the mid-IR to THz region^3^, compared with hyperbolic plasmon polariton materials, which are used in the UV to visible region.

The hyperbolic material platforms mentioned above have led to the development of a wide field of applications. The important areas are discussed in this Review by Lee et al., and we would like to highlight a few of them.

Conventional optics is fundamentally governed by the diffraction limit. Although waves with large wavevectors can carry the information of subwavelength features, they decay exponentially in vacuum for $\left| {\vec k} \right| > k_0$ due to the limited spatial bandwidth^11^. Therefore, an extensive scientific effort was invested in the development of hyperlenses that can overcome these limitations. Evanescent waves that are collected close to the imaged object can be turned into propagating waves with reduced wavevector^12^. Fig. 1i illustrates a hyperlens consisting of a curved multilayer HMM with an inner and outer radius. When light propagates through the lens, the product of the tangential component of $\left| {\vec k} \right|$ and the radius remains constant. If $\left| {\vec k} \right| \le k_0$ is given, light exits the lens as a propagating wave capable of transferring subwavelength information.

Anomalous diffraction, including negative refraction, has been widely studied in HMMs and HMSs. In conventional materials the Poynting vector $\vec S$ is parallel to $\vec k$ for transverse magnetic (TM) modes of light. However, for light propagating along an interface between an isotropic and a hyperbolic material, $\vec S$ and $\vec k$ are no longer parallel and negative refraction can occur. Figure 1j shows numerical results for a HMS silver grating with a flat dispersion relation at 543 nm^13^. Besides negative refraction, other exotic photonic effects are studied in HMM like the spin Hall effect of light. It refers to typically subwavelength spin-dependent displacements of light beams at interfaces^14^. HMMs have proven to be good candidates for enhancing this effect with a reported polarization-dependent beam-splitting of up to 165 µm for the visible light^15^.

Enhancing the interaction between light and matter is one of the essential advantages of nanophotonics. HMMs are well-suited to enhancing the spontaneous emission rates of emitters either within the material, or close to their surface. Due to their open isofrequency contour, HMMs have a diverging local density of photonic states (LDOSs). Following Fermi’s golden rule, the rate for spontaneous emission is proportional to the LDOS which makes HMMs ideal candidates for broadband enhancement of spontaneous emission. Figure 1k compares the Purcell factor (a measure for the spontaneous emission rate) of an emitter close to a metal surface and to a HMM^16^. Around the SPP resonance of the metal surface the Purcell factor increases strongly, however, this enhancement is spectrally narrow. For the multilayer HMM consisting of alternating layers of silver and silicon, multiple plasmon polariton bands broaden the bandwidth, and a high Purcell factor is achieved over the whole visible spectrum. By tuning the layer thickness and metallic filling factor of the HMM the plasmon modes can be readily controlled.

In summary, this Review by Lee et al.^1^ introduces nanophotonic concepts of hyperbolic materials with the fundamental principles necessary for an insightful understanding, and presents nanofabrication and characterization details essential to experimentalists. Some future challenges and perspectives in the field are discussed as well, offering important guidance for future work.
