# Correction: Nanophotonics shines light on hyperbolic metamaterials

**DOI:** 10.1038/s41377-022-00719-6

**Published:** 2022-01-26

**Authors:** Andreas Aigner, Judith M. Dawes, Stefan A. Maier, Haoran Ren

**Affiliations:** 1 Chair in Hybrid Nanosystems, Nanoinstitute Munich, Faculty of Physics, Ludwig-Maximilians-University Munich, Munich, 80539 Germany; 2 MQ Photonics Research Centre, Department of Physics and Astronomy, Macquarie University, Macquarie Park, NSW 2109 Australia; 3 Department of Physics, Imperial College London, London, SW7 2AZ UK

**Keywords:** Metamaterials, Micro-optics

Correction to: *Light: Science & Applications*

10.1038/s41377-021-00688-2, published online 10 January 2022

Following publication of this article^1^, it was noted that this article contains some errors. The order of Fig. 1c, d was reversed. Figure 1 has been updated in this Correction.

**Fig. 1:**
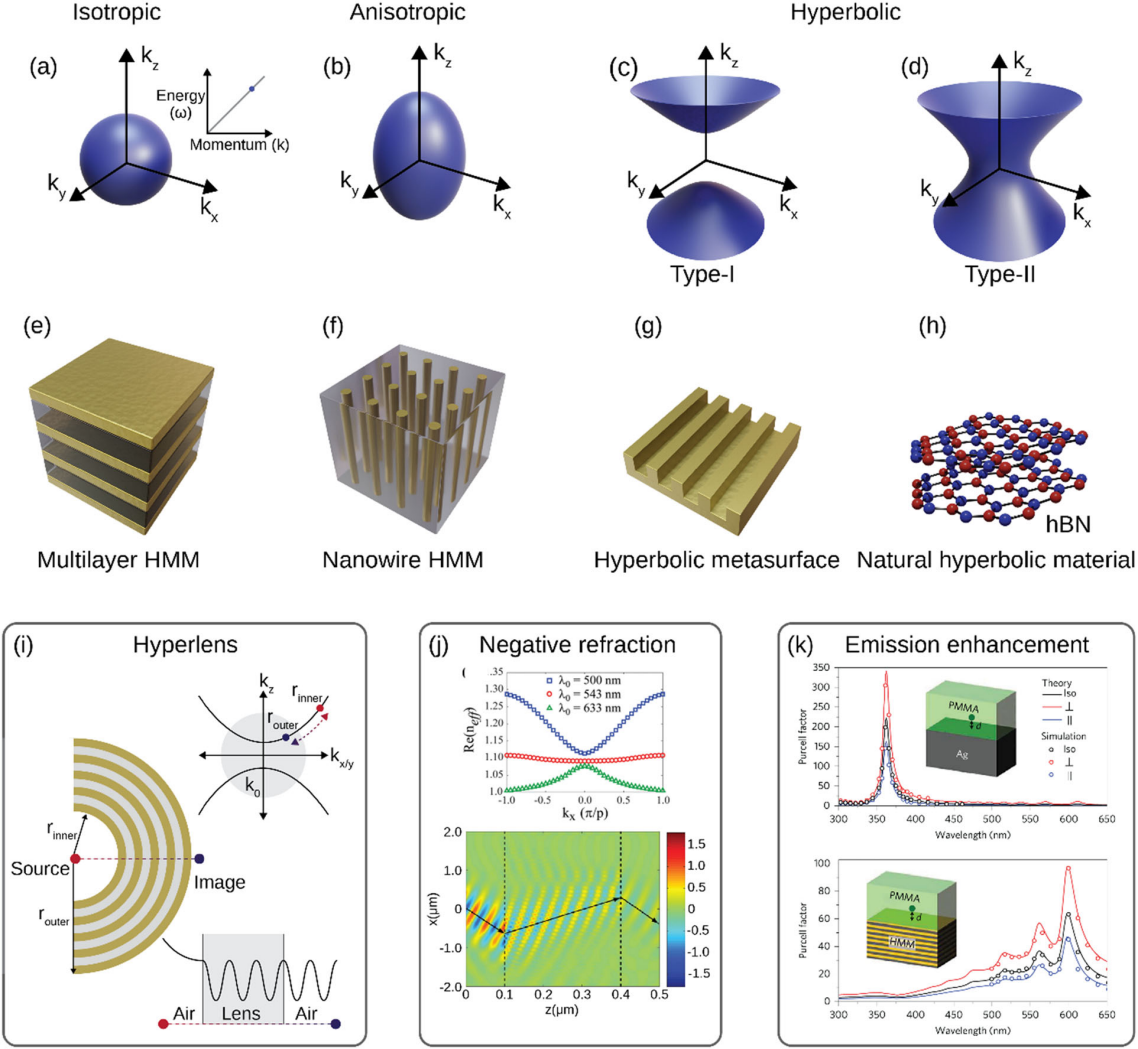
Principles and applications of hyperbolic metamaterials. Isofrequency contour for **a** isotropic, **b** anisotropic media, and **c** Type-I and **d** Type-II hyperbolic materials. Different hyperbolic material designs: **e** multilayer hyperbolic metamaterial (HMM), **f** nanowire HMM, **g** hyperbolic metasurface, and **h** natural hyperbolic material. Different applications of HMMs: **i** a HMM hyperlens, **j** negative refraction in a plasmonic grating HMS, and **k** spontaneous emission enhancement above a silver surface (top) and a HMM (bottom). **i**, **j**, and **k** are adapted from refs. ^3,13^, and ^16^, respectively

The original article has been updated as well.
